# Supplementary material for: HIVprotI: an integrated web based platform for prediction and design of HIV proteins inhibitors
Source: J Cheminform. 2018 Mar 9;10:12. doi: 10.1186/s13321-018-0266-y (PMC5845081; doi:10.1186/s13321-018-0266-y)
Supplement: Supplementary file 2 — Additional file 2. Source code of HIVProtI web server. [file 13321_2018_266_MOESM2_ESM.zip › HIVprotI_Source-code/hivprotI/weka-3-6-6/doc/weka/gui/GenericObjectEditor.GOETreeNode.html]

GenericObjectEditor.GOETreeNode


---


|  |  |  |
| --- | --- | --- |
| **Overview** \| **Package** \| **Class** \| **Tree** \| **Deprecated** \| **Index** \| **Help** \| **Weka's home** | |  |
| **PREV CLASS**   **NEXT CLASS** | **FRAMES**    **NO FRAMES**     **All Classes** |  |
| SUMMARY: NESTED \| FIELD \| CONSTR \| METHOD | DETAIL: FIELD \| CONSTR \| METHOD |  |


---


## weka.gui Class GenericObjectEditor.GOETreeNode

```
java.lang.Object
  javax.swing.tree.DefaultMutableTreeNode
      weka.gui.GenericObjectEditor.GOETreeNode
```

**All Implemented Interfaces:**: java.io.Serializable, java.lang.Cloneable, javax.swing.tree.MutableTreeNode, javax.swing.tree.TreeNode

**Enclosing class:**: GenericObjectEditor

---

``` public class GenericObjectEditor.GOETreeNode extends javax.swing.tree.DefaultMutableTreeNode ```

A specialized TreeNode for supporting filtering via Capabilities.

**See Also:**: Serialized Form

---

| **Field Summary** | |
| --- | --- |
| `static java.lang.String` | `MAYBE_SUPPORT`             color for "maybe support". |
| `static java.lang.String` | `NO_SUPPORT`             color for "no support". |

**Fields inherited from class javax.swing.tree.DefaultMutableTreeNode**

`EMPTY_ENUMERATION`


**Constructor Summary**

`GenericObjectEditor.GOETreeNode()`
  
          Creates a tree node that has no parent and no children, but which
allows children.

`GenericObjectEditor.GOETreeNode(java.lang.Object userObject)`
  
          Creates a tree node with no parent, no children, but which allows
children, and initializes it with the specified user object.

`GenericObjectEditor.GOETreeNode(java.lang.Object userObject,
boolean allowsChildren)`
  
          Creates a tree node with no parent, no children, initialized with the
specified user object, and that allows children only if specified.


| **Method Summary** | |
| --- | --- |
| `java.lang.String` | `toString()`             returns a string representation of this treenode. |

**Methods inherited from class javax.swing.tree.DefaultMutableTreeNode**

`add, breadthFirstEnumeration, children, clone, depthFirstEnumeration, getAllowsChildren, getChildAfter, getChildAt, getChildBefore, getChildCount, getDepth, getFirstChild, getFirstLeaf, getIndex, getLastChild, getLastLeaf, getLeafCount, getLevel, getNextLeaf, getNextNode, getNextSibling, getParent, getPath, getPreviousLeaf, getPreviousNode, getPreviousSibling, getRoot, getSharedAncestor, getSiblingCount, getUserObject, getUserObjectPath, insert, isLeaf, isNodeAncestor, isNodeChild, isNodeDescendant, isNodeRelated, isNodeSibling, isRoot, pathFromAncestorEnumeration, postorderEnumeration, preorderEnumeration, remove, remove, removeAllChildren, removeFromParent, setAllowsChildren, setParent, setUserObject`

**Methods inherited from class java.lang.Object**

`equals, getClass, hashCode, notify, notifyAll, wait, wait, wait`

**Field Detail**

### NO\_SUPPORT

```
public static final java.lang.String NO_SUPPORT
```

:   color for "no support".

    **See Also:**: Constant Field Values

---


### MAYBE\_SUPPORT

```
public static final java.lang.String MAYBE_SUPPORT
```

:   color for "maybe support".

    **See Also:**: Constant Field Values


**Constructor Detail**

### GenericObjectEditor.GOETreeNode

```
public GenericObjectEditor.GOETreeNode()
```

:   Creates a tree node that has no parent and no children, but which
    allows children.

---


### GenericObjectEditor.GOETreeNode

```
public GenericObjectEditor.GOETreeNode(java.lang.Object userObject)
```

:   Creates a tree node with no parent, no children, but which allows
    children, and initializes it with the specified user object.

    **Parameters:**: `userObject` - an Object provided by the user that constitutes the node's data

---


### GenericObjectEditor.GOETreeNode

```
public GenericObjectEditor.GOETreeNode(java.lang.Object userObject,
                                       boolean allowsChildren)
```

:   Creates a tree node with no parent, no children, initialized with the
    specified user object, and that allows children only if specified.

    **Parameters:**: `userObject` - an Object provided by the user that constitutes the node's data: `allowsChildren` - if true, the node is allowed to have child nodes -- otherwise, it is always a leaf node


**Method Detail**

### toString

```
public java.lang.String toString()
```

:   returns a string representation of this treenode.

    :   **Overrides:**: `toString` in class `javax.swing.tree.DefaultMutableTreeNode`
    :   **Returns:**: the text to display


---


|  |  |  |
| --- | --- | --- |
| **Overview** \| **Package** \| **Class** \| **Tree** \| **Deprecated** \| **Index** \| **Help** \| **Weka's home** | |  |
| **PREV CLASS**   **NEXT CLASS** | **FRAMES**    **NO FRAMES**     **All Classes** |  |
| SUMMARY: NESTED \| FIELD \| CONSTR \| METHOD | DETAIL: FIELD \| CONSTR \| METHOD |  |


---
